# Irregular Dietary Habits with a High Intake of Cereals and Sweets Are Associated with More Severe Gastrointestinal Symptoms in IBS Patients

**DOI:** 10.3390/nu11061279

**Published:** 2019-06-05

**Authors:** Clara Nilholm, Ewa Larsson, Bodil Roth, Rita Gustafsson, Bodil Ohlsson

**Affiliations:** 1 Department of Internal Medicine, Lund University, Skåne University Hospital Malmö, SE-20502 Malmö, Sweden; clara.nilholm@med.lu.se (C.N.); ewa-larsson@outlook.com (E.L.); bodil.roth@med.lu.se (B.R.); 2 Department of Gastroenterology and Hepatology, Skåne University Hospital Malmö, SE-20502 Malmö, Sweden; rita.gustafsson@med.lu.se

**Keywords:** irritable bowel syndrome, dietary habits, malnutrition, starch, sucrose, diet

## Abstract

Dietary advice constitutes one of the first choices of treatment for irritable bowel syndrome (IBS). We have recognized an increased prevalence of sucrase-isomaltase *(SI)* gene variants in IBS patients, possibly rendering starch- and sucrose-intolerance. The aims were to examine participants’ dietary habits at baseline, to correlate habits with gastrointestinal (GI) symptoms and blood levels of minerals and vitamins, and to examine the effect of a starch- and sucrose-reduced diet (SSRD) on GI symptoms. In the study 105 IBS patients (82 women, 46.06 ± 13.11 years), irritable bowel syndrome-symptom severity scale (IBS-SSS)>175, were randomized to SSRD for 2 weeks or continued ordinary eating habits. Blood samples, visual analog scale for irritable bowel syndrome (VAS-IBS), IBS-SSS, and 4-day food diaries were collected at baseline and after 2 weeks. Patients with irregular dietary habits exhibited higher IBS-SSS than patients with regular habits (*p* = 0.029). Women already on a diet had lower ferritin levels than others (*p* = 0.029). The intervention led to 66.3% of patients being responders, with differences in the change of IBS-SSS (*p* < 0.001), abdominal pain (*p* = 0.001), diarrhea (*p* = 0.002), bloating and flatulence (*p* = 0.005), psychological well-being (*p* = 0.048), and intestinal symptoms’ influence on daily life (*p* < 0.001), compared to controls. Decreased intake of cereals and sweets/soft drinks correlated with decreased scores.

## 1. Introduction

Irritable bowel syndrome (IBS) is a common disease without known etiology [1]. Different mechanisms such as an altered gut microbiota, low-grade inflammation, psychological factors, and yet undefined food intolerances have been discussed as potential factors in the pathophysiology of functional, non-organic gastrointestinal (GI) disorders. Gluten sensitivity is a newly recognized syndrome where patients with typical symptoms of IBS experience aggravated GI symptoms after intake of gluten, although celiac disease has been ruled out [2]. Similarly, many IBS patients also experience aggravated symptoms after milk intake, even when lactose intolerance cannot be confirmed [1].

Dietary advice constitutes one of the first choices of treatment for IBS. The National Institute for Heath and Care Excellence (NICE) guidelines, which advocate regular small meals, avoidance of gas-producing vegetables, a limited intake of fibers, and reduced coffee and tea intake, have been used for several years to alleviate IBS symptoms [3]. More recently, the low fermentable oligo-, di-, mono-saccharides and polyols (FODMAP) diet with avoidance of fermented carbohydrates has been established as a treatment for IBS [4]. However, almost 50% of the IBS population do not improve their symptoms following NICE recommendations or a low FODMAP diet [4,5]. 

Starch is an important energy source which can be found in cereals, legumes, and roots, accounting for 70–80% of the consumed calories worldwide [6]. Confectioneries and sugary drinks contain both refined sugar and starch [6]. The sucrase-isomaltase (*SI*) genes are responsible for a high percentage of the sucrase-, isomaltase-, and maltase activity in the brush border of the enterocytes [7]. Recently, we have recognized an increased prevalence of rare *SI* pathogenic variants in patients with IBS [8].

Our hypothesis was that dietary patterns with high intakes of cereals and sweets/soft drinks, containing high amounts of starch and sucrose, are associated with GI symptoms in an IBS population. To test this hypothesis, 105 IBS patients from the southernmost district of Sweden were enrolled in a prospective dietary intervention study. In the study, participants randomized to the dietary intervention group reduced their intake of starch and sucrose for 2 weeks, whilst those randomized to the control group continued with their normal diet. Blood samples were collected, and questionnaires about GI symptoms and a 4-day food diary were completed at study start and after 2 weeks. The primary aim of the present study was to examine the dietary habits at baseline, and to correlate the dietary habits with GI symptoms and blood levels of minerals and vitamins. Second, we wanted to examine the effect of a starch- and sucrose-reduced diet (SSRD) on GI symptoms.

## 2. Material and Methods

The study was approved by the Ethical Review Board of Lund University (2017/171) and performed in accordance with the declaration of Helsinki at Skåne University Hospital, Malmö, Sweden. All subjects gave their written, informed consent before inclusion in the study. The study was registered at ClinicalTrials.gov database (NCT03306381).

### 2.1. Study Design

The study design is a randomized, open clinical trial with a dietary intervention during two weeks (Figure S1). Patients with the IBS diagnosis set by their ordinary physician were contacted via mail and thereafter by telephone. After agreeing to participate in the study, patients completed a study questionnaire addressing socioeconomic factors and lifestyle habits and filled out a health declaration. A food diary during day 6–10 of the run-in period was recorded. Patients also completed questionnaires regarding GI symptoms; the Rome IV questionnaire, Irritable Bowel Syndrome-Symptom Severity Scale (IBS-SSS) and the Visual Analogue Scale for Irritable Bowel Syndrome (VAS-IBS). At the first appointment, the questionnaires were studied and subjects who suffered from abdominal pain at least once weekly along with altered bowel habits and had a score of ≥175 of the IBS-SSS, without any exclusion criteria (Figure S2), were included in the study. After randomization to a dietary intervention with reduced starch and sucrose, written and oral information about the SSRD was given. Patients randomized to serve as controls were informed to keep their ordinary food habits during the observational time, and were informed that they should receive all information about the diet after completion of the study. Blood samples were drawn and plasma and sera were separated. Whole blood samples, serum, and plasma were kept frozen in −20 °C or −80 °C, until further analyzed. The dietary regimen was kept for 2 weeks. At the end of the intervention, participants once again completed the food diaries (days 10–14), as well as IBS-SSS and VAS-IBS questionnaires (day 14). 

### 2.2. Patients

Inclusion criteria for the study were a diagnosis of IBS, age 18–70 years, and Northern European heritage; i.e., both parents and grandparents had to be born in Scandinavia or Northern Europe. Exclusion criteria were insufficient symptoms, <175 scores on IBS-SSS, presence of any organic GI disease, severe organic and psychiatric diseases, or already on a diet, i.e., vegan diet, gluten-free diet, low carbohydrate high fat (LCHF) diet, or low FODMAP diet (Figure S2). 

Patients were recruited from primary health care centers (PCC) in the southernmost district of Sweden as well as from a tertiary health care center, the Department of Gastroenterology and Hepatology. A patient registry provided by Region Skåne which registered all subjects who had received an IBS diagnosis code of K580 or K589 (according to the International Statistical Classification of Diseases and Related Health Problems–ICD-10) in a PCC during 2015–2017. Another registration of all patients who had received the diagnosis IBS, K580 or K589, from the Department of Gastroenterology and Hepatology during 2016–2017 was provided. 

In total, 2034 IBS patients from a PCC were identified. After exclusion of duplicates, 1039 patients remained. Invitation letters were sent to 528 patients after exclusion of all patients with names suggesting an ethnicity outside Scandinavia/Northern Europe, patients living outside the closest neighborhood of the cities Lund and Malmö, or patients whose telephone numbers could not be found. From the tertiary care center, 789 patients were identified. After exclusion of duplicates, 640 patients remained. Invitation letters were sent to 151 patients according to the same criteria as stated above. The patients were contacted in a random order via mail, with a letter detailing overall information about the study aim and design. A couple of weeks later the patients were contacted by phone and were, after receiving further information, asked to participate. Patients who were willing to participate (*N* = 145; 112 patients (77%) from PCC; 34 males (23%)) were sent a package of study questionnaires to complete prior to an appointment at the Internal Medicine Research Group, Skåne University Hospital, Malmö. Reasons not to participate are shown in Supplementary Table S1. After acceptance to participate, 40 patients (28%) whereof 11 were males (28%), were not enrolled in the study because they did not show up or were not willing to participate at a later time point (*n* = 18), had mild symptoms (*n* = 14), wrong diagnoses (*n* = 5) or were already on a diet in the exclusion criteria (*n* = 3). Thus, 105 patients (23 males (22%)) were finally included in the study (77 patients (73%) from the PCC) from the 679 invitation letters sent (15% inclusion rate). Of these, 98 participants completed the study (Figure S1). 

### 2.3. Dietary Advice

The patients were instructed to hold a diet with starch and sucrose restriction, according to the advice given to patients with congenital sucrase-isomaltase deficiency (CSID) [9]. Briefly, all forms of sucrose-containing food, e.g., candies, cakes, jam, and juice, were to be avoided. The content of starch should be reduced with less intake of cereals, but more intake of meat, fish, vegetables, fruits, egg and dairy products. The content of gluten and lactose in the ingested products were un-restricted. Fiber-rich bread, raw rice and fiber-rich pasta were preferred instead of white bread and ordinary rice and pasta, to delay the nutrient transport through the GI tract. Adding fat (e.g., avocado, olive oil) and/or protein to starch-rich foods was also recommended in order to further delay GI transport and enhance starch tolerance through longer exposure to digestive intestinal enzyme activity (such as isomaltase and sucrase) in the small intestine. In general, however, participants were still recommended to restrict their intake of fiber-rich cereals to a maximum of one serving per day. The patients were encouraged to eat slowly and chew their food properly in order to increase the secretion of amylase, which can contribute to degradation of starch. Candies and cakes should be replaced with nuts in the case of sweet cravings. Lists of suitable fruits and vegetables with less starch content were given (Supplementary Tables S2 and S3). The only advice encouraged was regarding starch and sucrose restrictions, not to reduce their energy content or their overall dietary habits regarding meal frequencies or regularity. The participants received a diagram over different kinds of carbohydrates and their digestive enzymes to explain the differences between various carbohydrates and which nutrition components to avoid. The participants could reach the study staff by telephone or email, whenever they wanted during the study. 

### 2.4. Questionnaires

#### 2.4.1. Study Questionnaire

A study questionnaire about sociodemographic factors, family history, lifestyle habits, medical health, pharmacological treatment, as well as subjective suffering from pain and discomfort were completed prior to study start. 

#### 2.4.2. Food Diaries 

All liquid and solid food intake, and time points of intakes, was registered during 4 days (Wednesday to Saturday), prior to study start and at the end of the intervention. At the same time, the participants had to register all types of GI symptoms, and at which time points they occurred, to be able to analyze associations between food intake and GI symptoms.

#### 2.4.3. Rome IV Questionnaire

The Rome IV questionnaire is developed to diagnose functional gastrointestinal disorders (FGID) [10]. Questions No 40–48 in the Swedish version of the questionnaire were used, after having received a license from The Rome Foundation, Inc. Raleigh, NC, USA.

#### 2.4.4. Irritable Bowel Syndrome-Symptom Severity Scale (IBS-SSS)

IBS-SSS consists of four questions regarding abdominal pain, abdominal distension, satisfaction with bowel habit and the impact of bowel habits on daily life, answered on visual analogue scales (VAS), where scores close to 0 mm suggest “no symptoms”, and scores close to 100 mm suggest “severe symptoms”. In addition, there is a question about the number of days with abdominal pain in the last 10 days [11]. The maximum achievable score is 500. Scores of 75–174 suggest mild disease, 175–299 suggest moderate disease and ≥300 suggest severe disease [11]. A responder to the dietary intervention is defined as a study participant experiencing ≥50 reduction of total IBS-SSS score registered at baseline. In addition, the prevalence of participants experiencing ≥50% reduction of total IBS-SSS score was also calculated.

#### 2.4.5. Visual Analog Scale for Irritable Bowel Syndrome (VAS-IBS)

The VAS-IBS is a validated questionnaire used to investigate GI symptoms [12]. The items measured on the VAS-IBS are abdominal pain, diarrhea, constipation, bloating and flatulence, vomiting and nausea, psychological well-being, and intestinal symptoms’ influence on daily life. The items are measured on a scale from 0 mm to 100 mm, where 0 mm represents a lack of symptoms and 100 mm very severe symptoms. The values are inverted from the original format, where lack of symptoms was set to 100. The questionnaire was also validated to measure changes of symptoms over time [13]. The values of VAS-IBS in a healthy control population were determined by a control material consisting of 52 healthy women (57.8%) and 38 healthy men (42.2%), mean age 43.3 ± 12.0 years, included from staff at the hospital.

#### 2.4.6. Laboratory Analyses

Ferritin, iron, total iron binding capacity (TIBC), folic acid, and cobalamin in plasma, and 25-hydroxy (25-OH) vitamin D in serum, were measured according to clinical routines at the Department of Clinical Chemistry. Reference values at the laboratory were used for classification of normal or abnormal values [14]. Transglutaminase antibodies in serum were analyzed by an enzyme-linked immunosorbent assay (ELISA) at the Department of Clinical Chemistry.

### 2.5. Analyses

#### 2.5.1. Categorization

All 4-day food diaries were thoroughly reviewed. A daily intake of three meals (breakfast, lunch, and dinner) with the addition of at most three snacks, ingested at regular time points, were considered as regular eating habits. Irregular eating habits were categorized as one or more of the following: (1) less than three meals each day; (2) more than six meals each day; or (3) irregular time points of food intake. Intake of water and coffee/tea without milk or sugar was excluded from the number of meal intakes, but was categorized as a snack when sugar and/or milk was added. Pizza or burger intake at least once during the 4-day run-in was registered as regular consumption of these dishes. Intake of sugar-rich soda or sugar-free soda was registered separately, and registered as the presence of intake versus no intake. The numbers of intakes during the 4 days of each type of soda were registered. Any type of on-going diet was registered. The food items were categorized into seven groups: meat including all kinds of meat and sausage; fish and seafood; vegetables, legumes, and roots; fruits, berries, nuts, almonds, peanuts, and seeds; dairy products; cereals; and sweets/soft drinks including candies, cakes, ice-cream, and sugar-rich soda. The daily frequency of food intake from each category was calculated, and the number of days with intake was registered. The total number of servings from each category over the 4-day registration period was then calculated. 

#### 2.5.2. Statistical Analyses

Two hypotheses were raised: (1) dietary patterns have effects on GI symptoms and nutritional status in IBS patients, and (2) reduced dietary intake of starch and sucrose improves GI symptoms in the patient cohort. No power analysis was performed since studies of SSRD in IBS have not been performed previously. However, in a previous nutrition study carried out by our research team where patients with type 2 diabetes received a carbohydrate-reduced diet, 23 patients were enough to demonstrate improved GI symptoms [15]. The distributions of values were tested by Kolmogorov–Smirnov test. Age, body mass index (BMI) and disease duration were normally distributed, and calculations to examine differences in these variables between groups were performed by Student’s *t*-test. Some of the GI symptom scores and laboratory analyses were not normally distributed, why these parameters were calculated by Spearman’s correlation test and/or Mann–Whitney U test. When borderline significance was present in normally distributed variables, calculations with Student’s *t*-test were added. Linear regression was used on logarithmic, continuous variables when adjustment for age and/or sex was necessary. Comparisons of dichotomous variables were calculated by Fisher’s exact test. When comparing GI symptoms before and after dietary intervention, Wilcoxon’s test was used. The difference between 2 weeks and baseline was calculated, and delta values were compared between groups. Values are presented as number and percentage, mean ± standard deviation, or median and interquartile range (IQR). Two-sided tests were used and *p* < 0.05 was considered statistically significant. All calculations were performed in SPSS (version 24; IBM Corporation) and performed as the intention to treat.

## 3. Results

### 3.1. Basal Characteristics

In total, 105 patients (82 women, 78.1%) were included. Mean BMI was 21.67 ± 3.94 kg/m^2^ (range 13.04–33.23 kg/m^2^). Basal characteristics are shown in Table 1. There were no differences in GI symptoms when accounting for level of education (university degree or lower) or level of physical activity (more or less than 60 min/week) (data not shown). 

Mean disease duration was 19.88 ± 13.77 years. Of the patients, 57 subjects (54.3%) had heredity for IBS. Thirty-seven subjects (35.2%) reported a trigger factor instigating disease development, most often emotional trauma (*n* = 20; 19.0%), infection (*n* = 5; 4.8%), or travel abroad (*n* = 2; 1.9%). The most common concomitant diseases and drug treatments are shown in Supplementary Table S4.

### 3.2. Gastrointestinal Symptoms

After classification according to Rome IV, 37 subjects (35.2%) suffered from mixed IBS (IBS-M), 26 subjects (24.8%) suffered from diarrhea-predominant IBS (IBS-D), 20 subjects (19.0%) suffered from constipation-predominated IBS (IBS-C), and 3 subjects (2.9%) suffered from unspecified IBS (IBS-U). All patients reported abdominal pain at least once weekly, but lack of a close association between the pain and altered bowel habits (i.e., two or more of the following at least in 30% of the time; pain associated with improvement or worsening with defecation, changed consistency of stool or changed frequency of defecation) in 17 patients (16.2%) rendered the diagnosis unspecified FGID when strictly following the Rome IV criteria [10]. Forty-eight subjects had moderate symptoms according to total IBS-SSS scores, whereas 55 subjects (52.4%) hade severe disease. 

The GI symptoms were markedly increased compared to healthy controls (Table 2). The only symptom which correlated to age was vomiting and nausea (*r* = 0.267, *p* = 0.006). The degree of constipation correlated with BMI (*r* = 0.282, *p* = 0.005), and differed between women and men (54 (28–72) mm versus 1 (0–56) mm, *p* = 0.003). Impaired psychological well-being correlated most of all with the intestinal symptoms’ influence on daily life (*r* = 0.331, *p* = 0.001), with weak correlations with constipation and bloating and flatulence (Table 2).

The foods most often associated with GI symptoms, as reported in the 4-day food diaries, were bread (both gluten-free and gluten-containing) (*n* = 31), candies or cakes (*n* = 26), coffee (*n* = 11), pizza and/or burger (*n* = 10), pasta (*n* = 8), cabbage (*n* = 7), fruits (*n* = 6), onion (*n* = 5), and beans (*n* = 4) (*n* = number of patients who registered GI symptoms). The symptoms most often reported were abdominal pain (*n* = 41), bloating (*n* = 33), flatulence (*n* = 19), and diarrhea (*n* = 11). 

### 3.3. Laboratory Data 

None of the participants expressed antibodies against transglutaminase in serum. CRP was elevated in 18.1% of the participants (Table 2).

Plasma levels of iron were below normal range, and total iron-binding capacity was elevated in around 10% of participants (Table 2). Ferritin levels correlated with age (*r* = 0.335, *p* = 0.001) and were higher in men than in women (235.30 (140.20–310.00) µg/L versus 55.98 (32.38–92.12) µg/L, *p* < 0.001). Patients adhering to an on-going diet had lower levels of ferritin than those without any dietary regimen (*p* = 0.004), a difference which was no longer significant after adjustment for age and gender (beta = −1.768, *p* = 0.080). When examining only women, the ferritin levels were lower in those women adhering to an on-going diet than those without (55.97 (28.41–82.85) µg/L versus 73.42 (44.20–114.20) µg/L, *p* = 0.042), also after adjustment for age (beta = −2.220, *p* = 0.029).

Twenty-three patients (22.1%) had levels of 25-OH vitamin D below 50 nmol/L, the limit for treatment recommendation with vitamin D supplementation in Sweden [16] (Table 2), even though many of them ingested vitamin D-containing drugs (Supplementary Table S4). Vitamin D levels were generally higher in individuals included in the autumn compared to individuals included in the spring (65.50 (57.00–79.25) mm versus 55.68 (47.00–72.16) mm, *p* = 0.018). Out of the 23 patients with low vitamin D levels, 6 patients were enrolled in the autumn and 17 patients were enrolled in the spring. 

### 3.4. Dietary Habits

About half of the patients had irregular dietary habits, eating either ≤2 meals per day, or more frequently, eating ≥7 meals per day (Table 3). Many patients did not consume complete meals but rather had small amounts of food every hour or every second hour. The day-to-day variation of food intake was generally low, which rendered limited intake of an all-round diet. Patients with irregular dietary habits exhibited more severe GI symptoms, especially in the case of bloating and flatulence, whereas blood levels of vitamins and minerals were unaffected by regularity (Table 4). Married participants were more prone to have regular dietary habits than subjects who lived alone (*p* = 0.027).

Eighty-eight patients (83.8%) from the study cohort had previously tried a diet. The diets most often tested were: a lactose-free diet (*n* = 49; 47.6%), a gluten-reduced diet (*n* = 32; 31.1%), a low FODMAP diet (*n* = 12; 11.6%), a vegetarian diet (*n* = 17; 16.5%), a LCHF diet (*n* = 6; 5.8%), or a fiber-and protein reduced diet (*n* = 3; 2.9%). At the time point for inclusion in the study, 69 patients (70.0%) were on a current diet. More women than men were on any on-going diet (*p* = 0.041) (Table 3).

During the four-day registration of the run-in period, 47 subjects (44.9%) ate pizza and/or burger at least once (*n* = 32), and four subjects ate it three or more times. In addition, they ate hot dogs, kebab, and other fast food, which were not included in the calculations, due to more seldom consumption. Men ingested fast food more often than women (Table 3). 

Eighteen subjects (17.1%) drank sugar-rich soda (most often Coca-Cola) and 14 subjects (13.3%) drank sugar-free soda during the four-day registration. A majority consumed soda once or twice (n = 11), and 7 subjects consumed soda three or more times during the run-in period. Eight patients drank sugar-free soda once or twice, whereas six drank sugar-free soda more than three times. In addition to soda, some patients drank other sweet beverages such as juice, cider, and chocolate milk. Patients who drank sugar-rich soda regularly had higher scores of total IBS-SSS (*p* = 0.026), vomiting and nausea (*p* = 0.039), and intestinal symptoms’ influence on daily life (*p* = 0.040), compared to those who never drank soda. The frequency of sugar-rich soda consumption during the run-in period correlated with the scores of total IBS-SSS (*r* = 0.250, *p* = 0.020), vomiting and nausea (*r* = 0.216, *p* = 0.045), and intestinal symptoms’ influence on daily life (*r* = 0.234, *p* = 0.029).

When examining dietary patterns, it could be seen that participants did not eat fish, vegetables and legumes and fruits very frequently. In contrast, they often ingested cereals and sweets/soft drinks (Table 5). Cereals were not only ingested in the form of bread, as frequent use of pasta and pancakes was also observed. The meat ingested was most often chicken, turkey, and forcemeat; seldom beef or meat from wild animals. Although participants adhering to a lactose-free diet drank lactose-free milk, some of them could still ingest milk and milk powder in foods such as white bread, potato chips and processed dishes, whereas hard cheese, which does not contain lactose, was excluded or seldom ingested. 

### 3.5. Effect of Dietary Advice

The total cohort was divided into a control group who continued with ordinary food habits, and an intervention group who started with the SSRD. There were no differences in sex (22 women/3 men versus 60 women/20 men; *p* = 0.242), age (41.4 ± 14.5 versus 47.5 ± 12.4 years; *p* = 0.064) or BMI (20.4 ± 3.9 versus 22.1 ± 3.9 kg/m^2^; *p* = 0.064) distribution between the control and intervention group. The dietary intakes in the control and intervention groups were similar at baseline (Table 5). 

Although the only advice given regarded reduction of starch and sucrose intake, more patients in the intervention group had regular dietary habits after 2 weeks than at baseline (*p* < 0.001). The change in regularity of diet correlated with the change in number of times they ingested cereals (r = 0.213, *p* = 0.038). As intake of cereals and sweets/soft drinks was reduced, intakes of fish, vegetables and legumes, fruits, and dairy products were increased, compared to the controls (Table 5). Several of the participants increased their intake of nuts and almonds instead of fruits and berries, which were included in the same category. 

**Figure 1:**
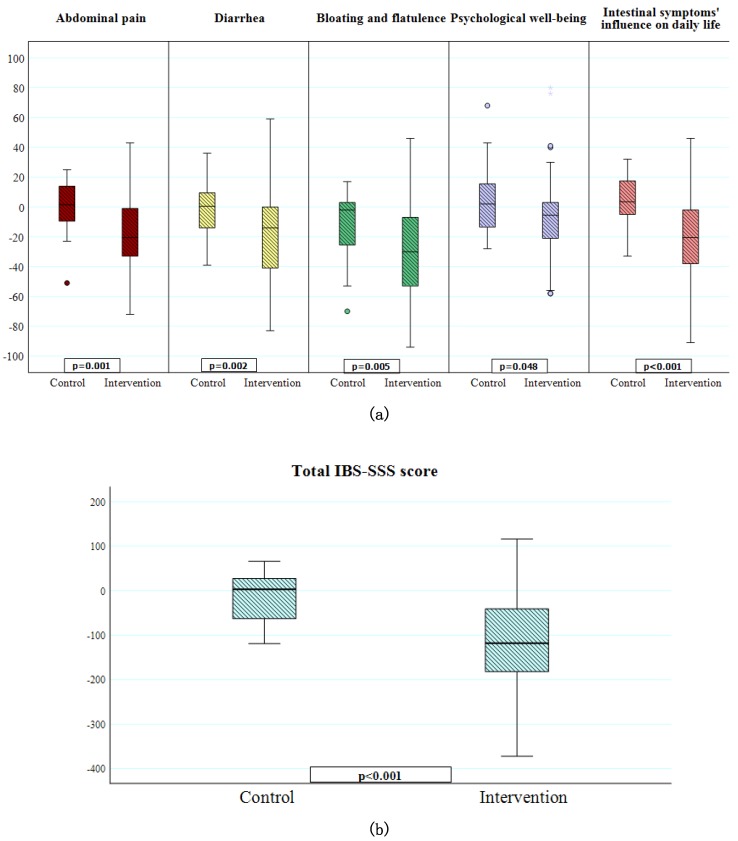
Changes in symptoms after the two-week dietary intervention. (**a**) Differences in the change of visual analog scale for irritable bowel syndrome (VAS-IBS) symptom scores (delta values) between controls (*n* = 25) and the intervention group (*n* = 80) regarding abdominal pain, diarrhea, bloating and flatulence, psychological well-being and intestinal symptoms’ influence on daily life. A score of 100 indicates the highest level of GI symptoms, very poor psychological well-being and high influence of symptoms on daily life. Missing values in two controls and five from the intervention group. Mann-Whitney U test. *p* < 0.05 was considered statistically significant. (**b**) Difference in the change of total irritable bowel syndrome-symptom severity scale (IBS-SSS) scores (delta values) between controls (*n* = 25) and the intervention group (*n* = 80). Missing values in two controls and five from the intervention group. Mann-Whitney U test. *p* < 0.05 was considered statistically significant.

In the control group, no differences in GI symptoms were observed between baseline and 2 weeks. In contrast, all GI symptoms were markedly reduced within the intervention group (Table 6). There were significant differences in the change of GI symptoms (delta values) between controls and the intervention group regarding total IBS-SSS score, abdominal pain, diarrhea, bloating and flatulence, psychological well-being and intestinal symptoms’ influence on daily life (Figure 1a,b). Fifty-three subjects (66.3%) in the intervention group were classified as responders, whereas a corresponding decrease of total IBS-SSS score in the control group was found in five subjects (20%) (*p* < 0.001). If the limit for responding had been set to a 50% reduction of total IBS-SSS, 25 subjects (31.1%) in the intervention group were classified as responders, and no one in the control group. The presence of moderate or severe disease, as classified by the IBS-SSS score, could not predict if a participant would be a responder (*p* = 0.397). The change in frequency of cereal intake correlated with lower IBS-SSS (*r* = 0.315, *p* = 0.002), less abdominal pain (*r* = 0.250, *p* = 0.016), and less diarrhea (*r* = 0.281, *p* = 0.007), and tended to correlate with less bloating and flatulence (*r* = 0.201, *p* = 0.055). The change in frequency of sweets/soft drinks intake correlated with lower IBS-SSS (*r* = 0.244, *p* = 0.019), less abdominal pain (*r* = 0.228, *p* = 0.029), less diarrhea (*r* = 0.218, *p* = 0.036), and less bloating and flatulence (*r* = 0.250, *p* = 0.016). 

Participants who experienced improved GI symptoms with the dietary intervention tended to have an immediate effect, which could usually be seen within 2–3 days after starting on the diet. Some of the participants experienced aggravated GI symptoms due to increased fiber intake, why they had to adjust fiber intake. Of those who had tested low FODMAP previously, they thought that SSRD was more efficient than low FODMAP, and the diet was easier to adhere to. 

## 4. Discussion

The main findings of the present study were that patients with IBS to a large extent had irregular dietary habits with a high intake of fast and processed food, cereals, and sweets/soft drinks, and a low intake of fish, vegetables, legumes, fruits, berries, and dairy products, not in line with the recommendations of a healthy Nordic diet [17]. Subjects with irregular dietary habits had more severe GI symptoms and women with an on-going diet had lower levels of ferritin. A majority of the patients had serum levels of 25-OH vitamin D below reference values. Introduction of the SSRD markedly improved GI symptoms.

The SSRD has been developed to treat patients with CSID [9,18]. This is the first study to our knowledge, which examines the effect of the SSRD in patients with IBS. The mechanisms underlying GI symptoms, and the improvement observed after the SSRD, have to be examined, in order to determine which individuals would respond to diet recommendations. It is well-known that undigested carbohydrates lead to gas production and osmosis in the bowel, which leads to an increased volume with distention of the bowel and induction of several GI symptoms [19,20,21]. The symptom development after intake of carbohydrates may depend on an overloading of the digestive system, surpassing the limit for what the GI tract has physiological capacity to degrade, with an accumulation of undigested carbohydrates in the lumen. At the same time as the intake of starch and sucrose was decreased, most of the participants changed to more regular meals. The positive effect of the diet may depend not only on a decrease of cereals and sweets/soft drinks, but also due to an increase of more healthy food items and dietary patterns. Irregular meal patterns [22,23], as well as fast food and candies [24,25,26], have previously been shown to be associated with IBS.

A second explanation for GI symptoms observed in the current study may be aberrant gut microbiota composition. Several studies have shown that the modern Western diet is associated with dysbiosis, which may lead to development of various diseases [27]. Third, genetic deficiency of sucrase-isomaltase activity may explain GI symptoms in some participants. CSID is an autosomal recessive disease, which results in enzymatic deficiency and a non-existent to highly limited ability to digest sucrose and starch. The prevalence of CSID is estimated to be 0.2% in North American and European ancestry [28]. More recent studies suggest that heterozygotes for CSID or functional polymorphisms of the *SI* gene may cause GI symptoms in certain patients [7,8]. Those with hypomorphic variants of the *SI* gene have reduced efficacy of low FODMAP diet, which is not focused on reductions in starch and sucrose intake [29]. Thus, this genetic defect could be one cause of unclear GI symptoms in line with lactose intolerance and fructose intolerance [30], since the *SI* genes are responsible for a high degree of the enzymatic activity in the brush border of the enterocytes [7].

Many patients experience some relief of their GI symptoms after introduction of a gluten-reduced diet, but the etiology to gluten hypersensitivity is still unknown [2]. The improvement could be explained by a decrease in total consumption of cereals, since gluten-free products are more expensive and sometimes viewed as less appetizing than common products. Furthermore, when gluten is reduced in the diet, it often leads to reduction of fiber intake as well, which may also reduce GI symptoms in IBS patients [3]. Concomitantly, the intake of starch may be increased, since elimination of gluten leads to increased importance of starch, in order to provide the structure and texture otherwise provided by gluten. Starch is an important energy source from cereals, legumes and roots, and accounts for 70–80% of the consumed calories worldwide [6]. Most starch produced is found in candies, drinks, and processed foods [6], which are products consumed in high amounts in the modern Western society. Thus, replacement of gluten with starch, mainly from potato, corn, tapioca, rice, and wheat into bread, may explain why gluten-free bread still provokes GI symptoms, as observed in the present study. Starches are grouped into rapid, slow and resistant digestible starches. Starch from cereals is more rapidly digested than starch from legumes and tubers [31]. Amylase is sometimes added to gluten-free products to enable the starch reduction [32]. In the same way, small amounts of lipids are added to alter the properties of starch [6]. Similarly to gluten-free products, lactose-free products are more processed than lactose-containing dairy products. Thus, high consumption of gluten-free and lactose-free products might in some cases aggravate GI symptoms, instead of alleviating them, due to overloading of starch and sucrose in the bowel lumen from highly processed foods. 

The markedly positive effect observed in patients with type 2 diabetes after introduction of the Okinawan-based Nordic diet [33] and in patients with IBS considering NICE guidelines or FODMAP [5], as well as in the current study after SSRD, indicates that one of the most basal issues to improve health, is to change dietary habits in the population. In the present study, we describe dietary patterns and not only nutrition content, as usually is reported [5], which add important information about dietary patterns of interest. To record a food diary is therapeutic per se, since the patients themselves become aware of how and what they eat. Lifestyle habits should be considered as a potential causal or contributive factor to FGID, and monitoring these habits should be a natural first-line approach by physicians in order to improve symptoms in IBS patients. A good nutritional supply, with healthy food and regular exercise, must be properly evaluated before extensive examinations are performed. 

The greatest differences between NICE guidelines and SSRD are that NICE encourages the participants to eat small and regular meals, reduce their intake of coffee and tea whereas water and other fluid intake should be increased, limit the amount of fruits and artificial sweeteners, and eat more oat and oat products. A similarity is that both diets recommend the restriction of starch found in processed foods [3]. The greatest differences between the low FODMAP diet as compared to the SSRD is that the low FODMAP diet recommends reduction of gluten and lactose intake, in addition to different recommendations for fruits and vegetables; e.g., avocado and several types of berries should be avoided, whereas bananas, oranges, and potatoes are allowed [19,20]. Further, the three phases of the FODMAP diet—first with restriction of all FODMAP-containing foods, followed by reintroduction of specific food items, and subsequent FODMAP personalization—are not included in the SSRD [4], although the SSRD diet also encourages to increase one item at each time of deselected food [9]. The obstacles with the low FODMAP diet are that it is difficult to adhere to, rather expensive, and might increase the risk of malnutrition when excluding many food items [4]. 

The self-assessed psychological well-being of the participants, rather, reflected the extent of impact their intestinal symptoms exerted on daily life, more than their degree of GI symptoms. This is in line with previous research, describing how psychological factors affect pain processing [34]. The high percentage of subjects with elevated plasma levels of CRP, in the absence of infection, is in agreement with the anticipation that the Western lifestyle is associated with low-grade chronic inflammation [35]. The high percentage of participants with low levels of vitamin D may be serious, since IBS subjects often are young women, who already have other risk factors to develop osteoporosis. Notably, the summer/autumn in Sweden 2018 was very hot with unusually long periods of sunshine, which should have prevented low levels of vitamin D. One can speculate whether the low vitamin D levels depend on insufficient dietary intake and/or insufficient time outdoors. Ferritin was correlated with age, which may depend on blood loss during menstruation in younger women. The lower levels of ferritin seen in women on a current diet could be explained by exclusion of several food items, resulting in inadequate nutritional intake.

One of the strengths of the study is the intention-to-treat calculation. We chose to make the statistical calculations with each symptom instead of IBS subgroups. In the case of mixed IBS, the characteristics of diarrhea and constipation may be equalized, and thus no alterations/correlations may be found. 

A limitation of the present study is its short duration, only two weeks. A continuation of SSRD for a longer time point will be evaluated. The placebo effect may be of importance in the study, but use of a control group who received the same number of contacts and health care should eliminate the differences in placebo effect between groups. It is customary to evaluate participants’ compliance to the diet, but the improvement of symptoms support that the participants performed changes in their dietary habits. In the future, we will analyze the nutritional composition and portion sizes of the food consumed by each patient, but the primary aim of the present study was to evaluate dietary patterns. There may be a bias in the selection of patients, since patients who are aware of poor dietary habits and want to make a lifestyle change possibly are more prone to be enrolled in such a dietary intervention study. 

## 5. Conclusions

Dietary habits affect the experience of GI symptoms and blood levels of minerals and vitamins in patients with IBS. Patients with regular dietary habits exhibit less pronounced GI symptoms than patients with irregular habits. A diet with reduced starch and sucrose content with less intake of cereals and sweets has marked effect on reducing GI symptoms in these patients.

## Figures and Tables

**Table 1:** Basal characteristics in the study cohort.

Parameters	Patients *N* = 105
**Sex** (women/men) (*n*, %)	82 (78.1)/23 (21.9)
**Age** (year)	46.06 ± 13.11
**Body mass index (BMI)** (kg/m^2^)	21.67 ± 3.94
Missing value	7
**Education** (*n*, %)	
Not completed school	1 (1.0)
Primary school	4 (3.8)
Secondary school	23 (21.9)
Education at least 1 year after secondary school	23 (21.9)
University	52 (49.5)
**Occupation** (*n*, %)	
Working full time	53 (50.5)
99–51%	13 (12.4)
50%	6 (5.7)
Sick leave	5 (4.8)
Retirement	16 (15.2)
Unemployed	3 (2.9)
Student	5 (4.8)
Parental leave	1 (1.0)
**Marital status** (*n*, %)	
Single/living alone	41 (39.0)
Married/cohabitated	62 (59.0)
**Smoking habits** (*n*, %)	
Regular smoking	6 (5.7)
Irregular smoking	5 (4.8)
Ex-smoker	37 (35.2)
Never smoked	54 (51.4)
**Snuff habits** (*n*, %)	
Snuff user	13 (12.4)
Ex-snuffer	5 (4.8)
Never snuffed	85 (81.0)
**Alcohol standard glass/1week** (*n*, %)	
<1	46 (43.8)
1–4	40 (38.1)
5–9	12 (11.4)
10-14	3 (2.9)
≥15	2 (1.9)
**Strenuous exercise (min/week)**	
Never	11 (10.5)
<30 min	24 (22.9)
30–60 min	16 (15.2)
60–90 min	12 (11.4)
90–120 min	14 (13.3)
>120 min	26 (24.8)

Values from two subjects were missing in socioeconomic factors and lifestyle habits. Strenuous exercise means exercise that leads to increased heart rate.

**Table 2:** Values of gastrointestinal symptoms and blood samples at baseline.

Parameters	Values *N* = 105	Number of Patients Outside Reference Values	Correlation between Psychological Well-being and GI Symptoms
IBS-SSS total score	310 (248–353)		*r* = 0.203 *p* = 0.039
Abdominal pain Ref value: 5 (1–13)	51 (36–65)	103	*r* = 0.121 *p* = 0.223
Diarrhea Ref value: 3 (0–10)	55 (11–73)	103	*r* = 0.087 *p* = 0.380
Constipation Ref value: 6 (2–16)	48 (1–72)	96	*r* = 0.216 *p* = 0.028
Bloating and flatulence Ref value: 10 (2–23)	78 (63–87)	103	*r* = 0.237 *p* = 0.016
Vomiting and nausea Ref value: 2 (0–4)	15 (2–48)	101	*r* = 0.100 *p* = 0.316
Psychological well-being Ref value: 5 (2–15)	49 (24–69)	103	
Intestinal symptoms’ influence on daily life Ref value: 2 (0–14)	71 (52–839)	103	*r* = 0.331 *P* = 0.001
CR*P* (mg/L) Ref value: <3.0	0.72 (0.60–1.72)	19	
Iron (µmol/L) Ref value: 9–34	16.10 (11.25–20.83)	10 (9 below)	
Total iron binding capacity (µmol/L) Ref value: 47–80	63.99 (58.10–70.98)	11 (2 below)	
Ferritin (µg/L) Ref value: (13–148)	69.24 (41.58–139.32)	30 (8 below)	
Folic acid (nmol/L) Ref value: ≥6	13.88 (10.41–21.24)	4	
Cobalamin (nmol/L) Ref value: 150–500	364.20 (258.72–429.08)	16 (2 below)	
25-OH Vitamin D (nmol/L) Ref value: >75	61.00 (52.02–74.00)	<50: 23 (21.1%) <75: 81 (77.1%)	

CR*P* = C-reactive protein; IBS-SSS = irritable bowel syndrome-symptom severity scale. Individual gastrointestinal (GI) symptoms were measured in mm on visual analog scale for irritable bowel syndrome (VAS-IBS). Blood samples were analyzed in plasma except 25-hydroxy (OH) vitamin D which was analyzed in serum. Values from two subjects were missing in GI symptoms and one subject was missing in blood samples. Reference values of GI symptoms are from 90 healthy controls and the reference values of blood samples from the Department of Clinical Chemistry (14). Spearman’s correlation test. *P* < 0.05 was considered statistically significant.

**Table 3:** Differences in dietary patterns between women and men during the four-day run-in period.

	Women *N* = 82 (*n*, %)	Men *N* = 23 (*n*, %)	*p*-Value
Dietary habits			1.00
Regular habits	40 (48.8)	10 (43.5)	
Irregular habits	41 (50.0)	11 (47.8)	
Missing value	1 (1.2)	2 (8.7)	
Diet regimes			0.041
On a current diet	59 (72.0)	10 (43.5)	
Not on a current diet	23 (28.0)	11 (47.8)	
Missing value	1 (1.2)	2 (8.7)	
Pizza/burger intake			0.049
Intake	33 (40.4)	14 (91.3)	
No intake	48 (58.5)	7 (30.4)	
Missing value	1 (1.2)	2 (8.7)	
Soda intake			0.608
Sugar-rich	14 (17.1)	4 (17.4)	
Sugar-free	10 (12.2)	4 (17.4)	
No soda drinking	57 (69.5)	13 (56.5)	
Missing value	1 (1.2)	2 (8.7)	

Number (*n*) and percentage (%) of different dietary patterns. Values from one woman and two men were missing. Regular habits were defined as 3–6 meals/day, ingested at regular time points. Fisher’s exact test. *p* < 0.05 was considered statistically significant.

**Table 4:** Difference in gastrointestinal symptoms and laboratory analyses in regard to regular and irregular dietary habits during run-in.

Parameters	Regular Habits *N* = 50	Irregular Habits *N* = 52	*p*-Value
IBS-SSS total score	296 (236–350)	329 (260–387)	0.051 (0.029) *
Abdominal pain	49 (36–62)	56 (37–76)	0.081
Diarrhea	56 (10–75)	54 (10–72)	0.924
Constipation	40 (1–68)	64 (1–76)	0.118
Bloating and flatulence	74 (52–82)	84 (68–92)	0.006
Vomiting and nausea	7 (1–31)	24 (7–52)	0.041
Psychological well-being	46 (24–61)	54 (31–71)	0.194
Intestinal symptoms’ influence on daily life	70 (47–81)	70 (60–87)	0.052 (0.018) *
CRP (mg/L)	0.600 (0.6–1.495)	0.92 (0.60–2.64)	0.109
Iron (µmol/L)	16.40 (12.54–21.12)	16.36 (10.35–18.94)	0.244
TIBC (µmol/L)	61.43 (56.54–67.06)	65.30 (58.46–72.48)	0.116
Ferritin (µg/L)	88.11 (50.34–140.20)	60.84 (30.83–133.45)	0.044 (0.249) **
Folic acid (nmol/L)	14.40 (11.26–21.72)	13.68 (9.70–20.84)	0.239
Cobalamin (nmol/L)	368.70 (279.50–433.33)	339.05 (251.92–417.98)	0.408
25-OH Vitamin D (nmol/L)	61.33 (52.32–70.50)	59.30 (50.00–80.12)	0.627

CRP = C-reactive protein; IBS-SSS = irritable bowel syndrome-symptom severity scale; TIBC = Total iron binding capacity. Regular habits were defined as 3–6 meals/day, ingested at regular time points. Individual gastrointestinal (GI) symptoms were measured in mm on visual analog scale for irritable bowel syndrome (VAS-IBS). Blood samples were analyzed in plasma, except 25-hydroxy (OH) vitamin D which was analyzed in serum. Values are presented as median and interquartile ranges (IQR). Comparisons between groups were performed by Mann–Whitney U test. Calculations supplemented with: * = Student’s *t*-test. ** = linear regression, adjusted for age. *p* < 0.05 was considered statistically significant.

**Table 5:** The intake of different food items during run-in and after two weeks.

Categories	Control Group *N* = 25			Intervention Group *N* = 80			*p*-Value
	Times/ day	Days/ 4 days	Times/ 4 days	Times/ day	Days/ 4 days	Times/ 4 days	
**Baseline** Missing	1			2			
**Meat**	1 (1–2)	4 (1–4)	4 (1.25–8)	1 (1–2)	4 (2–4)	4 (2–8)	0.393
**Fish/seafood**	1 (0–1)	1 (0–2)	1 (0–1.75)	1 (0–1)	1 (0–2)	1 (0–2)	0.806
**Vegetable**/ **legumes**	1 (1–1.75)	3 (2–4)	4 (2.25–4)	1 (1–1)	4 (3–4)	4 (3–4)	0.302
**Fruits/nuts**	1 (1–1)	2 (2–4)	2 (2–4)	1 (1–1)	3 (1–4)	3 (1–4)	0.652
**Dairy products**	1 (1–1.75)	4 (2–4)	4 (2–4)	1 (1–2)	3 (2–4)	4 (2–8)	0.604
**Cereals**	2 (1–2)	4 (4–4)	8 (4–8)	2 (1–2)	4 (4–4)	8 (4–8)	0.504
**Sweets/** **soft drinks**	1 (1–2)	4 (1–4)	4 (2–8)	1 (1–2)	4 (2–4)	4 (2–8)	0.920
**After 2 weeks** Missing	3			6			
**Meat**	1 (1–2)	3 (2–4)	4 (2–7)	1 (1–1)	4 (2–4)	4 (2–4)	0.793
**Fish/seafood**	1 (0–1)	1 (0–1.5)	1 (0–1.25)	1 (1–1)	1 (1–2)	1 (1–2)	0.022
**Vegetable/** **legumes**	1 (1–1.25)	4 (2.5–4)	4 (2.5–5)	1 (1–2)	4 (4–4)	4 (4–8)	0.034
**Fruits/nuts**	1 (0.75–2)	2 (0.5–4)	2 (0.5–5)	2 (1–2.9)	4 (3–4)	8 (4–8)	<0.001
**Dairy products**	1 (1–1)	4 (3–4)	4 (3–4)	1 (1–2)	4 (3–4)	4 (3–8)	0.036
**Cereals**	2 (1–2)	4 (4–4)	8 (4–8)	1 (1–2)	4 (1–4)	4 (1–8)	0.002
**Sweets/soft drinks**	1 (0.75–2)	3 (0.5–4)	4 (0.5–4)	0 (0–1)	0 (0–2)	0 (0–2)	0.001

The control group continued with their ordinary food habits during the two-week observational time period. The frequency of each food intake/day was registered, as well as the number of such days during the four-day registration (day 6–10 during run-in and day 10–14 during the study). The total frequency of each item/4 days were compared between the two groups by Mann–Whitney U test. Values are presented as median and interquartile rages (IQR). *p* < 0.05 was considered statistically significant.

**Table 6:** The effect on GI symptoms of the dietary intervention.

GI Symptoms	Control		Intervention		*p*-Value Intervention
	Baseline	2 weeks	Baseline	2 weeks	
IBS-SSS total score	310 (247–351)	271 (238–325)	306 (250–356)	190 (118–282)	<0.001
Abdominal pain	49 (27–62)	48 (26–63)	52 (37–65)	29 (8–52)	<0.001
Diarrhea	47 (5–70)	33 (6–62)	57 (18–76)	18 (2–56)	<0.001
Constipation	54 (30–69)	28 (2–68)	47 (1–73)	22 (0–50)	<0.001
Bloating and flatulence	78 (68–89)	76 (54–87)	78 (60–85)	46 (12–61)	<0.001
Vomiting and nausea	29 (6–50)	20 (3–38)	11 (1–34)	4 (0–28)	0.003
Psychological well-being	47 (24–71)	49 (31–66)	50 (24–69)	41 (14–60)	0.002
Intestinal symptoms’ influence on daily life	68 (53–78)	68 (63–84)	72 (52–86)	49 (19–68)	<0.001
Missing value	0	5	2	6	

The control group continued with their ordinary food habits during the two-week observational time period. Comparisons before and after two weeks was performed by Wilcoxon test. No statistically significant differences were found before and after two weeks in the control group, wherefore, *p*-values are not shown. Values are presented as median and interquartile rages (IQR). *p* < 0.05 was considered statistically significant.

## Supplementary Materials

The following are available online at https://www.mdpi.com/2072-6643/11/6/1279/s1, Table S1: Reasons for not being eligible for/declining study participation, Table S2: Recommendations of fruit intake according to a starch- and sucrose- reduced diet, Table S3: Recommendations of vegetable and legume intake according to a starch- and sucrose-reduced diet, Table S4. Concomitant diseases besides irritable bowel syndrome (IBS) and drugs used as regular treatments, Figure S1: CONSORT 2010 Flow Diagram, Figure S2: Flow chart over inclusion and exclusion criteria.
