# Supplementary material for: Irregular Dietary Habits with a High Intake of Cereals and Sweets Are Associated with More Severe Gastrointestinal Symptoms in IBS Patients
Source: Nutrients. 2019 Jun 5;11(6):1279. doi: 10.3390/nu11061279 (PMC6627681; doi:10.3390/nu11061279)

Supplementary Table S1. Reasons for not being eligible for/declining study participation

**Reasons for non-inclusion**	**Number**
Did not answer when calling	188
Did not want to participate	157
Wrong adress	62
Already on a diet in the exclusion criteria	45
No or mild symptoms	37
Another diagnosis	27
Moved from the region	14
Pregnancy	4

Supplementary Table S2. Recommendations of fruit intake according to a starch- and sucrose- reduced diet

Well tolerated	Tolerated by some	Not tolerated
Avocado	Persimmons	Apples
Blackberries	Plums	Apricots
Blueberries	Raisins	Bananas
Boysenberries	Watermelon	Cantaloupe
Cherries		Dates
Cranberries		Grapefruits
Currants		Guava
Figs		Honeydew melon
Gooseberries		Mangos
Grapes		Nectarines
Kiwi fruits		Oranges
Lemons		Passion fruits
Limes		Peaches
Loganberries		Pineapples
Olives		Tangelos
Papayas		Tangerines
Pears		
Pomegranates		
Prunes		
Raspberries		
Rhubarbs		
Strawberries		

Information from Ref No 9.

Supplementary Table S3. Recommendations of vegetable and legume intake according to a starch- and sucrose-reduced diet

**Well tolerated**	**Tolerated by some**	**Not tolerated**
Alfafa sprouts	Edamame soybeans	Beets
Artichokes*	Jicamas	Black beans
Arugulas	Leeks	Black-eyed peas
Asparagus*	Okra	Butternut
Bamboo shoots	Pumpkins	Carrots
Bok choy	Snow peas	Cassavas
Broccoli*	Tempeh	Chickpeas
Brussels sprouts*	Tofu	Corn
Cabbages*	Yellow wax beans	Garlic
Cauliflower*		Green peas
Celery		Lentils
Chard		Kidney beans
Chicories		Lima beans
Chives		Navy beans
Collard greens		Onion
Cress		Parsnips
Cucumbers		Pinto beans
Eggplants		Potatoes
Endive		Soybeans
Green beans		Split peas
Kale		Sweet potatoes
Lettuces		Yams
Mung bean sprout		
Mushrooms		
Mustard green		
Peppers		
Radishes		
Spaghetti squash		
Spinach		
Tomatoes		
Turnips		
Yellow squash		
Zucchini		

Information from Ref No 9.

Supplementary Table S4. Concomitant diseases besides irritable bowel syndrome (IBS) and drugs used as regular treatments

**Diseases**	
Allergy	17
Hypothyroidism	12
Asthma bronchialis	11
Depression	11
Hypertension	10
Migraine	7
Lactose intolerance	5
Pain in back and joints	5
**Drug treatment**	
Antidepressants	21
Laxatives	14
Proton-pump inhibitor	13
Levothyroxine	13
Hormonal treatment	11
Vitamin D	11
Statins	8
Folic acid	6
Cobalamin	5
Inhaled asthma medication	5

Figure S1. CONSORT 2010 Flow Diagram

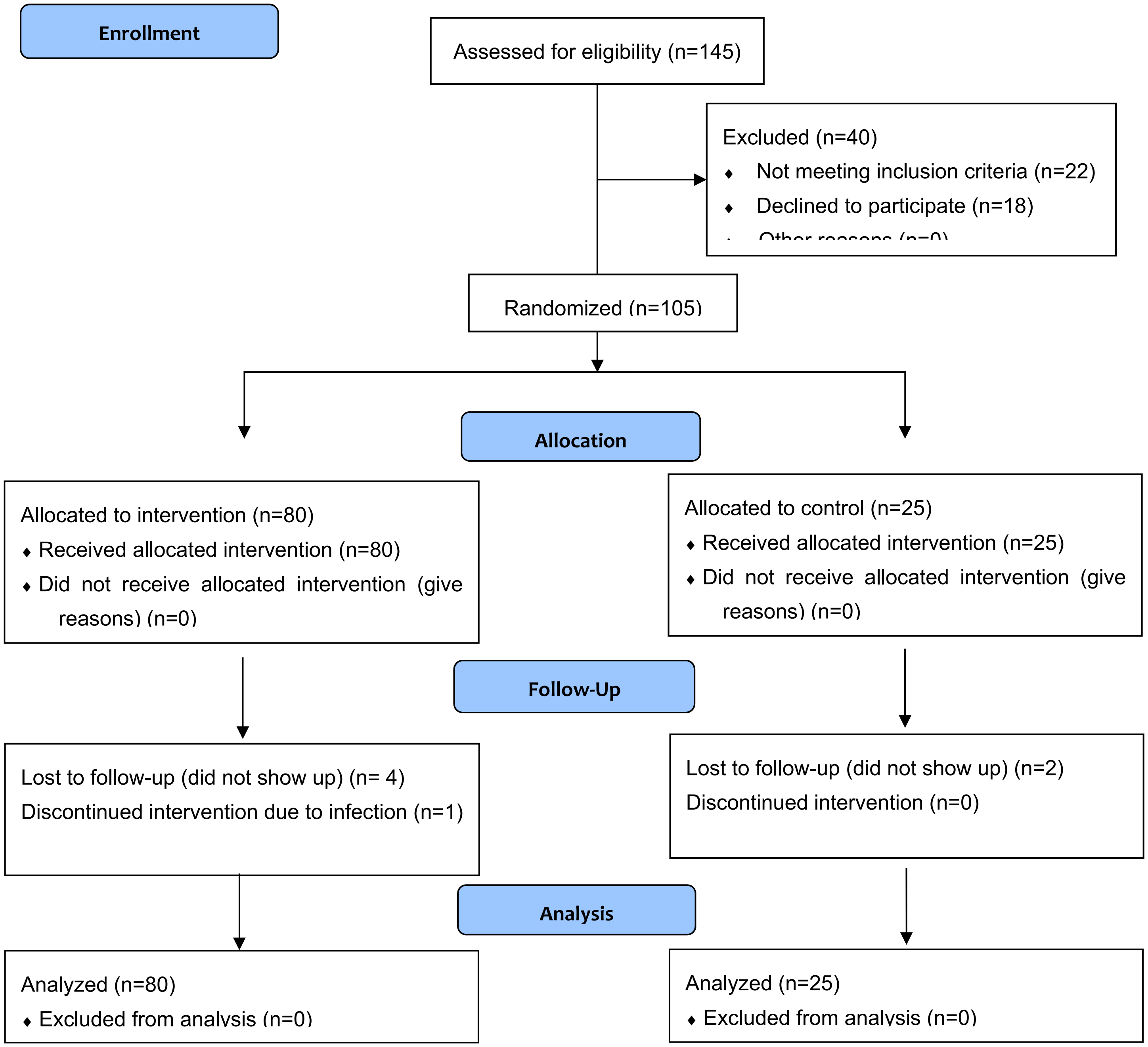

Figure S2. Flow chart over inclusion and exclusion criteria

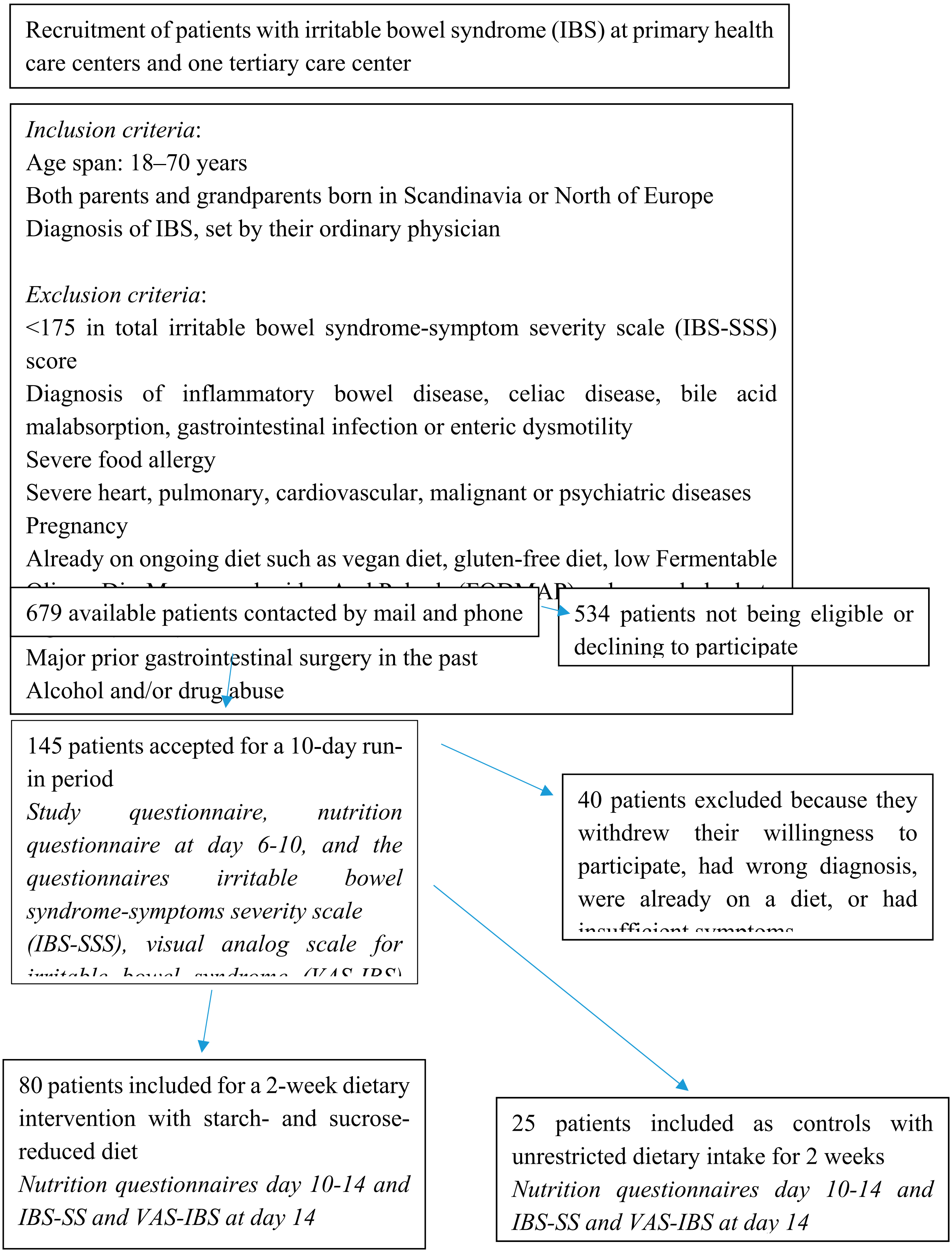

Supplement: Supplementary file 1 [file nutrients-11-01279-s001.pdf]
